# Comparison of Modified With Classic Morrow Septal Myectomy in Treating Hypertrophic Obstructive Cardiomyopathy

**DOI:** 10.1097/MD.0000000000002326

**Published:** 2016-01-15

**Authors:** Bangrong Song, Ran Dong

**Affiliations:** From the Department of Cardiac Surgery, Beijing Anzhen Hospital, Capital Medical University, Beijing, China.

## Abstract

This study aimed to compare the efficacy and safety of the classic Morrow septal myectomy with the modified procedure in treating hypertrophic obstructive cardiomyopathy (HOCM).

A retrospective study was conducted to compare the outcomes of classic with modified Morrow septal myectomy in 42 patients treated from January 2005 to July 2011. Preoperative and postoperative ventricular septal thickness, left ventricular (LV) outflow tract velocity and gradient were measured echocardiographically.

In both groups, the ventricular septal thickness, LV outflow tract velocity, and LV outflow tract gradient were significantly decreased after the operation. The modified Morrow procedure group, however, showed significantly greater reduction in these echocardiographic parameters than the classic procedure group. All patients in the modified procedure group were asymptomatic postoperatively with a postoperative transvalvular pressure gradient <30 mm Hg. In the classic procedure group, only 14 (87.5%) patients, however, were asymptomatic postoperatively with a postoperative transvalvular pressure gradient <30 mm Hg, and 2 patients still had severe LV outflow obstruction postoperatively.

The modified Morrow septal myectomy is safe and effective in treating HOCM patients, and is superior to the classic procedure in reducing the LV outflow tract gradient and velocity, restoring normal anatomic atrioventricular size, and alleviating symptoms associated with HOCM.

## INTRODUCTION

Hypertrophic obstructive cardiomyopathy (HOCM) is a primary heterogenetic disease of the cardiac myocardium. This disease is characterized by concentric and asymmetric cardiac hypertrophy, and increased thickness of cardiac walls, potentially complicated by abnormal left ventricular ejection fraction.^1^ Surgical myectomy has been the standard treatment for severely symptomatic HOCM patients for nearly five decades.^1^ There, however, is currently a great debate on the more appropriate treatment for HOCM between alcohol septal ablation and surgical myectomy.^2^

The classic Morrow operation (Morrow septal myectomy) was described for the first time in 1975.^3^ In the classic Morrow operation, cardiac obstruction is relieved by resecting relatively small (2–5 g) sections of muscle tissue in the proximal ventricular septum, which widens the outflow tract and decrease the flow drag (Venturi).^2^ Thus, systolic combined contact between the mitral valve and hypertrophied septum allow for an immediate gradient reduction that can relieve symptoms.^4^ Recently, many different modifications of the classic procedure have been proposed, aiming to reconstruct the left ventricular (LV) outflow with better outcomes by enlarging the resected area.^4–8^ The modified Morrow septal myectomy has been used in our hospital since 2005. There, however, is no concluded results in the efficacy comparison of the modified operations with the classic Morrow operation. This has caused more debate about the preferred treatments for HOCM between the alternative methods (ie, alcohol ablation) and surgical myectomy.

The current study was conducted to compare the efficacy and safety of the classic Morrow septal myectomy with the modified procedure in treating HOCM patients.

## MATERIALS AND METHODS

### Patients

This is a retrospective study including 42 HOCM patients treated with classic or modified Morrow septal myectomy from January 2005 to July 2011 at the Department of Cardiac Surgery of the Beijing Anzhen Hospital, China. There were 27 men and 15 women with a mean age of 45 ± 16 years. The study protocol was approved by the Department of Cardiac Surgery, Beijing Anzhen Hospital Affiliated to Capital Medical University, China.

Patients were included if then met the following criteria: diagnosed with HOCM^9^; age >18 years; exertional or nonexertional symptoms of dyspnea, syncope, or cardiac dysfunction; LV outflow ≥50 mm Hg; the ratio of ventricular septum to LV posterior wall >1.3:1; resting differential pressure of LV outflow >75 mm Hg.

### Clinical Data Collection

The clinical data and demographic information were extracted from the medical records of each patient, including age, sex, presence of preoperative systolic anterior motion (SAM), complete atrioventricular block, complete left bundle branch block, Alfieri technique, anterior mitral valve transversally folded shape, mitral valve replacement, coronary artery bypass grafting, and right ventricular outflow tract enlargement. Preoperative mitral insufficiency degree was determined by echocardiography^10^ and categorized as mild reflux (regurgitation jet area <4 cm^2^), moderate reflux (regurgitation jet area ≥4 and <8 cm^2^), and severe reflux (regurgitation jet area ≥8 cm^2^).

### Surgical Procedures

All septal myectomy operations were performed under general anesthesia with low temperature extracorporeal circulation. Continuous anterograde cold blood cardioplegia was used for myocardial protection. A proximal incision was made below the aortic valve to dredge the LV outflow. Mitral valves were managed by interatrial groove pathways. Operations were guided by transthoracic echocardiography and transesophageal echocardiography.

**FIGURE 1:**
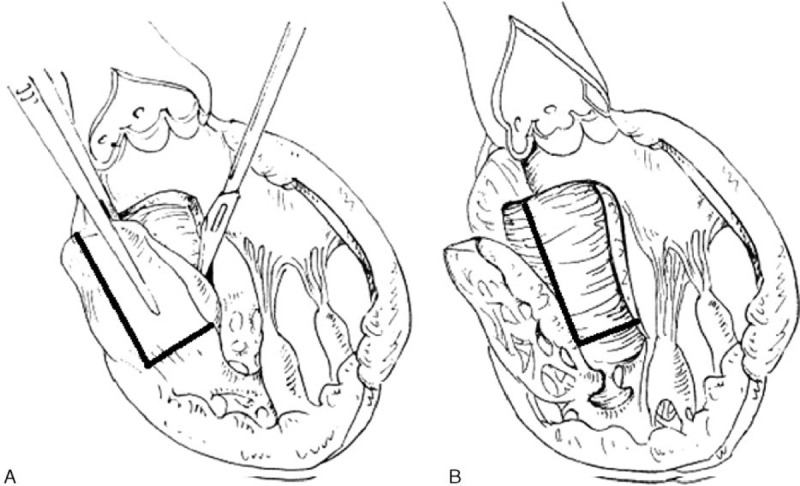
The resection areas in Morrow procedures were marked by the black lines. (A) The classic Morrow procedure. (B) The modified Morrow procedure.

For the classic Morrow septal myectomy procedure, a 2 to 3-cm incision was made from the midpoint of the right coronary leaflet to the left and right mitral-septal contacts, extending to the basal septum, as previously designed by Morrow et al (Fig. 1A).^3^ For the modified Morrow septal myectomy procedure, a 5 to 6-cm incision was made by extending the classic incision with a midventricular resection, from the ventricular septum (approximately 5 mm left to the right aortic valve) to the anterior mitral valve contact of the mitral valve (Fig. 1B). The incision was extended distally beyond the mitral–septal contact to the heart apex. Mitral valve repair or replacement was conducted simultaneously, restricting the mitral valve motion and relieving the subaortic obstruction and mitral regurgitation.

### Postperative Assessments

Ventricular septal thickness, LV outflow tract velocity, and LV outflow tract gradient were measured using transthoracic and transesophageal echocardiography. Special attention was paid to postoperative 1 week and postoperative 1 year. Transvalvular pressure gradient (TPG) was measured at postoperative 1 week.

### Statistical Analysis

Continuous variables are expressed as means ± standard deviations (SD), and categorical variables as frequencies or percentages. SPSS v.17 (SPSS, Inc., IBM, Chicago, Ill., USA) was used for the statistical analysis. Preoperative and postoperative data were compared using paired *t*-tests. Efficacy was compared between groups using independent samples *t*-tests. A *P*-value less than 0.05 were considered statistically significant.

## RESULTS

### General Characteristics of the Patients

Of the 42 HOCM patients, 16 (38.1%) patients were treated with the classic Morrow procedure and 26 (61.9%) with the modified Morrow procedure. There was no significant difference in the clinical characteristics between the 2 groups (Table 1).

**TABLE 1:**
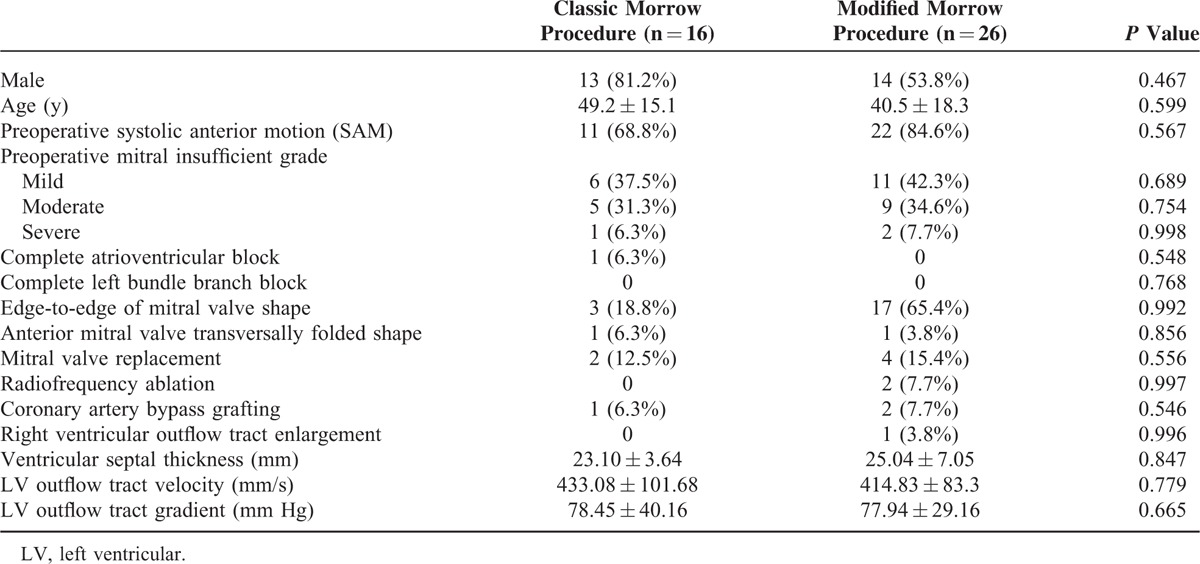
Demographic and Clinical Characteristics of the Patients (n = 42)

### Echocardiographic Results

In both groups, the ventricular septal thickness, LV outflow tract velocity, and LV outflow tract gradient were significantly decreased after the operation (Table 2). The modified Morrow procedure group, however, showed significantly greater reduction in these echocardiographic parameters than the classic procedure group, suggesting its superiority in improving the LV outflow tract gradient and velocity (*P* < 0.05, Table 3).

**TABLE 2:**
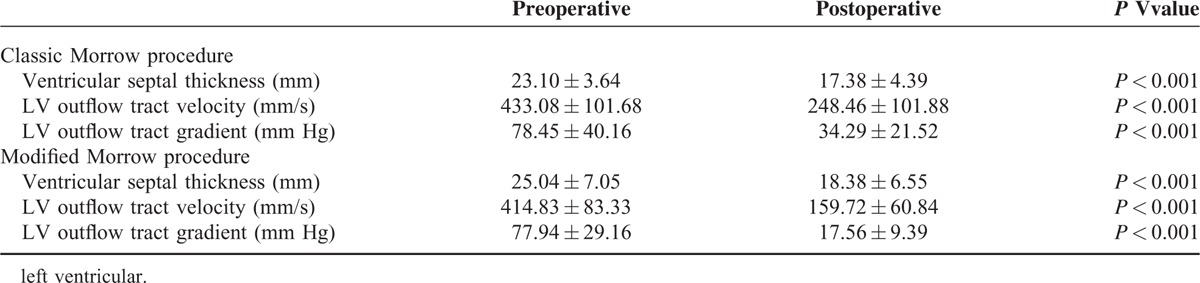
Preoperative and Postoperative Echocardiographic Parameters (n = 42)

**TABLE 3:**
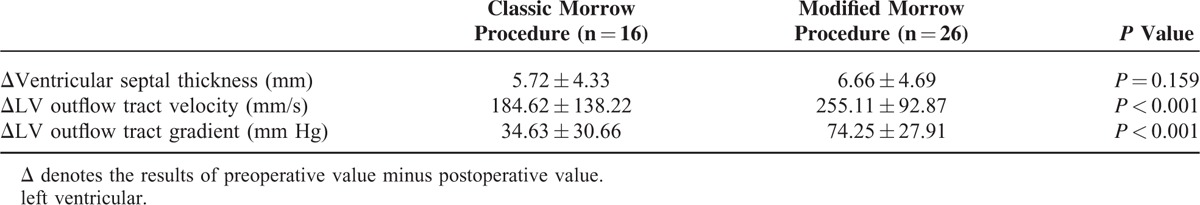
Comparison of Reduction in Echocardiographic Parameters Between the 2 Groups

### Efficacy Results

The patients were studied for a median period of 20 months. In the classic Morrow procedure group, 14 (87.5%) patients were asymptomatic postoperatively with a postoperative TPG < 30 mm Hg, and 2 (12.5%) patients showed LV outflow velocity of 411 mm/s with severe LV outflow obstruction (postoperative TPG > 90 mm Hg). One patient in the classic procedure group showed third-degree atrioventricular block. On the contrary, all 26 (100%) patients in the modified Morrow group were asymptomatic postoperatively with a postoperative TPG < 30 mm Hg. Two patients died from low cardiac output in the classic procedure group within 30 days and 1 year postoperatively, respectively. No patients died in the modified procedure group within 1 year postoperatively. No complications, such as heart failure and thromboembolism occurred in the modified procedure group.

## DISCUSSION

Classic and modified Morrow procedures have been used since 1961, and many variations of this procedure have been reported with varied efficacies.^11^ Gao et al^7^ reported a modified Morrow procedure than could achieve full mitral regurgitation, improve or eliminate SAM in all cases, which is consistent with the findings of our study. In addition, patients treated with the modified Morrow procedure were asymptomatic postoperatively, or only showed mild dyspnea but no syncope.^7^ Similarly, Dörge et al^8^ reported good early results in HOCM patients after extended surgical myectomy and reconstruction of the subvalvular mitral apparatus, with significantly decreased mortality and morbidity, and good functional capacity even in high risk patients with severe preoperative symptoms.^8^ Therefore, modified Morrow procedure demonstrated optimal results compared with the classic surgery, with potential long-term survival benefits and immediate alleviation of symptoms.

Based on the current understanding of HOCM mechanisms, SAM cannot be eliminated using incisions that only affect the aortic valve, even when the LV outflow is enlarged.^9^ This is because the abnormal blood flow remains, allowing symptoms to progress and raising the risk of interventricular septum perforation.^9^ Separation of papillary muscle can correct the abnormal connection between the musculi papillares and the front wall, posteriorly displace the mitral valve, and prevent abnormal outflow and blood flow driving force.^12^ In rare conditions, enlarged sail leaf valves may cause SAM symptoms to remain even after the treatment, which is an indication of folding the anterior mitral valve.^13^ Furthermore, Cooley et al^14^ suggested that folding the anterior mitral valve can reduce valve size and abnormal stresses, thus reducing the chordae tendineae and the valve slack. Mitral valve replacement, however, can eliminate symptoms of SAM at the cost of increasing the risk of severe artificial valve related complications.^15^ Thus, mitral valve replacement is only recommended for patients with mitral valve abnormalities, such as prolapse, calcification, or >18 mm length of anterior ventricular septum.^16^

Up to 66% of HOCM patients have structural abnormalities of the mitral valve, including increased leaflet area, leaflet elongation, or anomalous papillary muscle insertion into the anterior mitral leaflets. These conditions that can lead to death or incomplete remission of the obstruction if not recognized and managed.^17^ Thus, accurate assessment of the anatomic features is essential for selecting the proper and optimal treatment of HOCM patients. Notably, the wider incision area offered by the modified Morrow procedure may be more optimal for treating direct papillary muscle connection and fusion to the interventricular septum, achieving long-term symptom alleviation in a large portion of patients.^18^ Thus, the papillary muscle fusion zone should be separated, and isolation or incision is required in case of abnormal chordae tendineae or mitral valve fiber attachment to the ventricular septum or free wall.

In conclusion, the modified Morrow septal myectomy is safe and effective in treating patients with HOCM, and is superior to the classic Morrow procedure in reducing the LV outflow tract gradient and velocity, restoring normal anatomic atrioventricular size, and alleviating the symptoms associated with HOCM.
